# Acute normovolemic hemodilution in combination with tranexamic acid is an effective strategy for blood management in lumbar spinal fusion surgery

**DOI:** 10.1186/s13018-022-02950-8

**Published:** 2022-02-05

**Authors:** Yang Li, Yan Zhang, Xiutong Fang

**Affiliations:** 1 Department of Thoracic Surgery, The First Hospital of Jilin University, Changchun, 130021 China; 2 Department of Orthoapedic Surgery, Beijing Shijitan Hospital, Capital Medical University, NO.10, Tieyi Road, Haidian District, Beijing, 100038 China

**Keywords:** Lumbar spinal fusion surgery, Acute normovolemic hemodilution, Tranexamic acid

## Abstract

**Background:**

The retrospective study was designed to compare the effectiveness and safety of acute normovolemic hemodilution (ANH), tranexamic Acid (TXA), and a combination of ANH and TXA in lumbar spinal fusion surgery.

**Methods:**

Data of 120 patients underwent multi-level posterior spinal fusion for treating degenerative lumbar disease between June 2013 and December 2017 was collected, retrospectively. Four treatment strategies were enrolled, including ANH, TXA, a combination of ANH and TXA, and without any patient blood management. Intraoperative blood loss, hemoglobin and PCV at the end of surgery and at the postoperative first day, and postoperative drain collection, and intraoperative and postoperative transfusion and rate of transfusion were also collected.

**Results:**

Intraoperative blood loss and postoperative drain collection of the TXA group, ANH combined with TXA group were statistically lower than those in the control group and ANH group (*P* < 0.05). Intraoperative and postoperative transfusion amount and rate of intra-operative allogenic transfusion of the ANH group, TXA group, and ANH combined with TXA group were statistically lower than those of the control group (*P* < 0.05). Hemoglobin and PCV at postoperative the first day in the ANH group, TXA group, and ANH combined with TXA group were significant higher than those in the control group (*P* < 0.05). The combination of TXA and ANH group achieved the lowest intraoperative blood loss, postoperative drain collection and allogenic transfusion rate.

**Conclusion:**

A combination of TXA and ANH might be an effective strategy for reducing the rate of transfusion and blood loss in patients underwent lumbar spinal fusion surgery.

## Background

Lumbar spinal fusion surgery is designed to decrease pain generated from the degenerative vertebral segments by stopping the motion of the joint. During perioperative periods of the spinal reconstructive surgery, massive blood loss is inevitable and most patients in the surgery require allogeneic blood transfusion to avoid development of postoperative anemia, severe hypotension, metabolic acidosis, infections, acute lung injury, and cardiac arrest [1]. Although various blood-conservation interventions have been put forward, blood transfusion is still needed among lots of patients. Intraoperative and postoperative hemorrhage would cause the risks of infectious diseases transmission, postoperative infection, and immune modulation effects introduced by allogeneic blood, which are urgently needed to be solved [2–4]. In order to reduce bleeding during major spine surgery and achieve an optimal clinical outcome, various patient blood management strategies have been put forward to improve the clinical outcomes.

Several studies have evaluated the role of patient blood management strategies in blood loss and hemorrhage during and after spinal surgery [5–7], such as preoperative autologous blood donation, hypotensive anesthesia, acute hypervolemic hemodilution, acute normovolemic hemodilution (ANH), and intraoperative cell salvage system [8]. Acute hemodilution is easy to operate, less physiological interference with good effect and has become a common clinical blood saving method. ANH releases part of the intravascular volume before surgery, and reduces the preload, making it easier to obtain greater hemodilution effect, increasing the tolerance of blood loss, and protecting red blood cells. Carless et al*.* showed that ANH might recover up to 70% of the shed blood in orthopedic surgery and significantly reduce the use of erythrocyte [9]. Subsequently, lots of literatures demonstrated that ANH is an effective alternative strategy [10, 11].

In recent years, Tranexamic acid (TXA) has been used in spine surgery extensively to reduce perioperative and postoperative loss. The perioperative use of TXA was shown to prevent intraoperative blood loss and reduction the need for blood transfusions in numerous studies [12–14]. Raksakietisak et al. found 15 mg/kg TXA could significantly reduce blood loss and transfusion in low-risk adults undergoing complex laminectomy [15]. A meta-analysis conducted by Yang et al. demonstrated that patients receiving perioperative TXA had a reduction of blood loss along with a statistically significant decrease in the need for blood transfusion [14]. A common concern surrounding the use of perioperative TXA is its potential to induce thromboembolic events, such as deep vein thrombosis and pulmonary embolism. However, this particular risk is very low to negligible. Although many literatures had assessed the use of TXA or ANH in spinal surgery, the conclusion still remains inconsistency.

Thus, this study aimed to compare the effectiveness and safety of ANH, TXA, and a combination of ANH and TXA in reducing blood transfusion in lumbar spinal fusion surgery. The results of this study might help researchers improving the understanding of different blood management strategies.

## Methods

### Population and selection criteria

This retrospective study was approved by our hospital institutional review board before its commencement. The data of 120 patients (age range, 55–70 years) underwent multi-level posterior spinal fusion (≥ 2 levels) for treating degenerative lumbar disease between June 2013 and December 2017 were collected. Among the 120 patients, four types of patient blood management were included, including ANH group, TXA group, ANH combined TXA group, and control group (without patient blood management). Demographic information including age, sex, body mass index, height, duration of surgery, number of fused segments, and number of interbody fusion were collected.

The inclusion criteria included: (1) Patients aged 50–70 years old who were diagnosed as degenerative lumbar disease and without allergy to TXA; (2) Patients underwent multi-level posterior spinal fusion (≥ 2 levels) through a posterior midline approach; (3) Patients were treated with ANH, TXA, a combination of ANH and TXA, or without patient blood management. The exclusion criteria of patients enrolled included: (1) patients with liver cirrhosis, seriouscardiac disease, chronic renal failure, cancer, a history of thromboembolic disease (deep vein thrombosis, ischemic heart disease, pulmonary embolism, transient ischemic attack, strokes, or subarachnoid hemorrhage), bleeding disorders, hypercoagulation status, disseminated intravascular coagulation and pregnancy; (2) patients receiving antiplatelet and/or anticoagulant therapy at the time of the study; (3) patients with hematocrit < 35% and hemoglobin < 11 g/dL; (4) patients with abnormal coagulant function or vascular diseases before surgery; (5) patient with New York classification > grade II, or American society of anesthesiologists classification > grade II; and (6) patients with valvular heart diseases, arrhythmias, severe pulmonary, hepatic and renal dysfunction, tumor disease or metastasis and (7) patients underwent minimally invasive spine surgery.

### Surgical technique

**Fig. 1:**
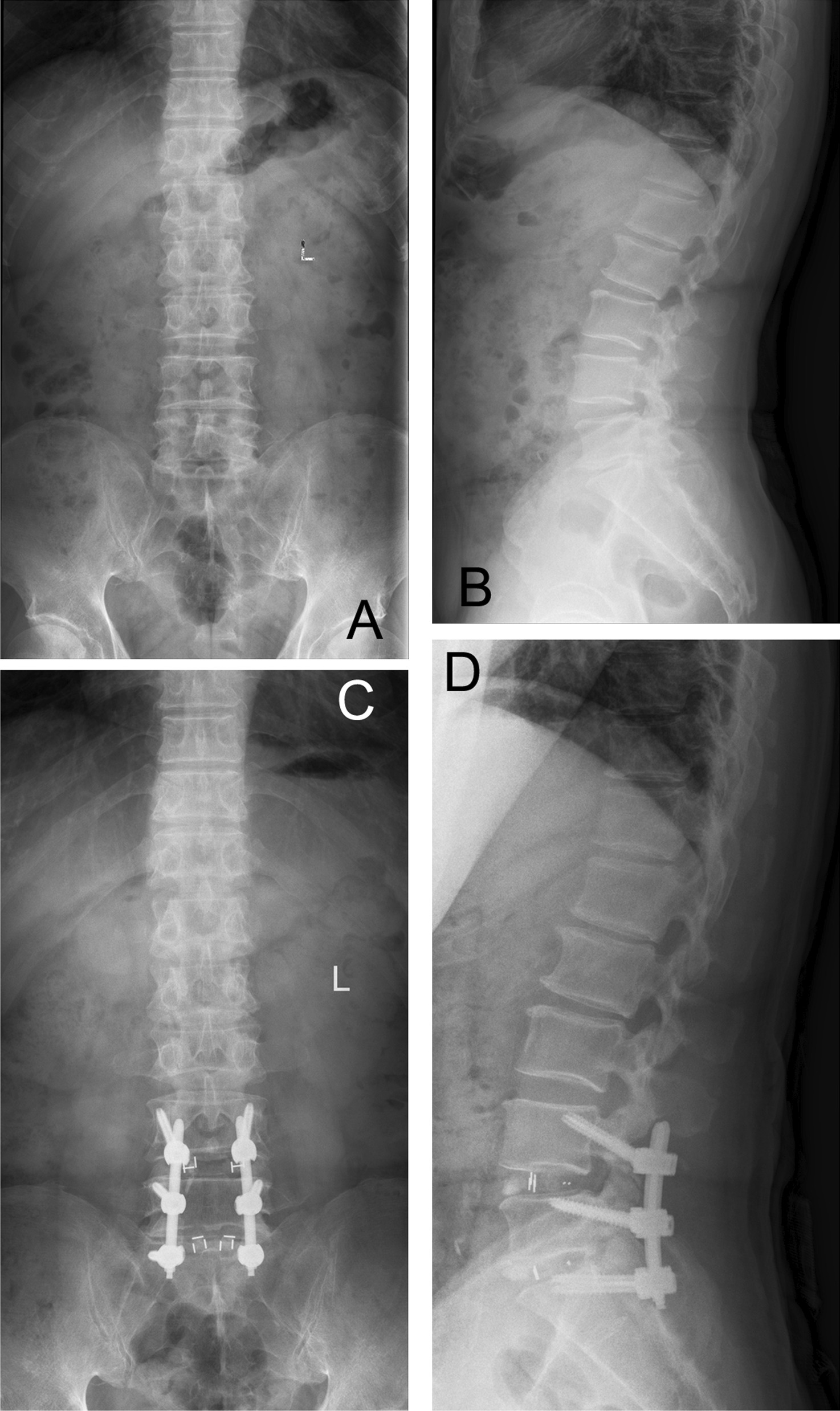
The preoperative and postoperative X-rays in a patient with lumbar spinal stenosis (L34, L45) underwent posterior laminar decompression, cortical bone trajectory internal fixation and interbody fusion. **A** The preoperative frontal X-ray. **B** The preoperative lateral X-ray. **C** The postoperative frontal X-ray. **D** The postoperative lateral X-ray

Both 300 U/kg erythropoietin and 500 mg/ml iron sucrose were applied per week for 4 weeks in patients with hemoglobin < 110 g/L in the preparation of operation. All patients underwent a similar operative technique of lumbar spinal fusion surgery. Patients were placed in a prone position on the operating table under general anesthesia. Through posterior midline skin incision, subperiosteal exposure of respective levels done. Pedicle screws were placed with free hand technique and connecting rods were installed. After resection of the laminae and removal of the corresponding disc transforaminally, a cage filled with autogenous bone graft was obliquely placed into intervertebral disc space. Radiography was taken to confirm the pedicle screws and cage position. After achieving hemostasis, negative pressure drainage was placed, and a layer to layer suture was carried out to close the wound. All wounds were closed with negative suction drain (Fig. 1).

### Blood management method

For patients in ANH group, intraoperative autologous transfusion was performed with the Elmed 500 (Medtronics, Denver, CO, USA). The intraoperative autologous transfusion device was used for each case, and blood would be returned to the patient if there was a sufficient volume to process. Hemodilution was accomplished by the removal of artery blood from femoral artery after induction and intubation. The approximate volume of blood withdrawn was determined based on the estimated blood volume, preoperative hematocrit, and desired hematocrit. The blood with anticoagulant was collected in a fenwal autologous blood collection kit (Baxter, Baxter health Corporation, Deerfield, IL, USA). The targeted bleeding volume was 10%-15% of total blood volume according to the formula: in male, blood volume = height × 28.5 + weight × 31.6–2820; and in female: blood volume = height × 16.25 + weight × 38.46–1369. Total blood volume = weight of blood volume/1.060 [13]. The collected blood was stored at 0–4 °C. The blood was replaced with 6% hydroxyethyl starch (130/0.4) in equal volume. After that, hypervolemic treatment was applied with 6% hydroxyethyl starch (130/0.4) and 10 ml/kg lactated ringer's solution (equal to 20% of total blood volume) within 30 min. Then, norepinephrine and phenylephrine were applied to maintain mean arterial blood pressure below 20% of the basal blood pressure. During the operation, autologous blood transfusion would be performed when hemoglobin was lower than 8–9 g/L or packed cell volume (PCV) < 24–27%, and homologous blood would be used when necessary. After surgery, iron supplementation was implemented with 500 mg/ml iron sucrose per week.

All patients in the TXA group were given a dose of 15 mg/kg of TXA (Transamin; Daiichi Pharmaceutical, Tokyo, Japan) before a skin incision was made, followed by 1 mg/kg/h infusion till skin closure [16].

Patients in the control group received neither ANH or TXA or any other anti-fibrinolytic drug during the intraoperative period.

### Outcome evaluation

#### Blood loss estimation

ABL is also referred to as maximum allowable blood loss as it marks the amount after which hemorrhage complications start occurring. ABL was calculated using the following formula:

$${\text{ABL}} = {\text{EBV}}*\left( {{\text{Hi}} - {\text{Hf}}} \right){\text{Hi}}.$$

where ABL indicates allowable blood loss, EBV indicates estimated blood volume, Hi indicates Initial hematocrit and Hf indicates final hematocrit;

Traditionally, EBV for an adult male = 75 mL/kg, EBV for an adult female = 65 mL/kg.

Blood transfusion would be needed if blood loss exceeded the ABL. At the end of the surgery, the blood soaked swabs were weighed using an electronic weighing scale. The amount of blood in the suction apparatus and the amount of saline were recorded. Total amount of blood loss was calculated using the following formula:

$$\begin{aligned} {\text{Total amount of blood loss}} & = \{ {\text{amount of fluid collected in suction apparatus}}\;({\text{mL}}) \\ & \quad {-}{\text{amount of saline}}\;({\text{mL}}) \\ & \quad + {\text{amount of blood content in weighed swabs}}\;({\text{mL}})\} . \\ \end{aligned}$$

where 1 g blood equaled 1 mL blood.

Blood products would be transfused postoperatively if the value of hemoglobin/PCV significantly dropped (< 8 g/d) and the drain collection increased (> 500 mL).

#### Hematological indicators

Blood routine indicators including hemoglobi, PCV, and platelets were determined by automated hematology analyzer (Beckman coulter, CA, USA). Blood routing indicators were tested preoperatively, at the end of surgery and at the postoperative first day.

#### Coagulation assay

Activated partial thromboplastin time (APTT) is recorded as the time required for the formation of a stable clot in seconds in citrated plasma, with addition of factor XII activator and CaCl2. Prothrombin time (PT) is a blood test that measures the time from the liquid portion of blood to clot in the presence of sufficient concentration of calcium and tissue thromboplastin. International normalized ratio (INR) is derived from PT which is calculated as a ratio of the patient’s PT to a control PT. APTT, PT and INR are hematological indices reflecting the coagulation status of patients. Blood was centrifuged at 3000 rpm for 15 min for testing of APTT and PT according to standard procedures. Coagulation assays were performed preoperatively.

#### Postoperative drain volume

Postoperative drain outputs including deep and superficial drains were collected and recorded.

#### Transfusion

Intraoperative and postoperative transfusion amount and rate of transfusion were collected.

### Statistical analysis

Continuous variables (including age, body weight, height, duration of surgery, body mass index number of fused segments, number of interbody fusion, hemoglobin, PCV, platelets, APTT, PT, INR, blood loss, drain, intraoperative allogenic transfusion amount and postoperative allogenic transfusion amount) were expressed as mean ± standard deviation, and categorical data (including gender, intraoperative rate of allogenic transfusion and postoperative rate of allogenic transfusion) were presented as proportion or percentage. Analysis of variance and pearson *χ*^2^ test were used to estimate the difference of study parameters between groups for continuous variables and categorical data, respectively. All analyses were carried out using SPSS 20.0 (IBM, Armonk, NY, USA). A *P* value < 0.05 indicated statistical significance.

## Results

### General results of enrolled patients

The baseline characteristics of enrolled patients were shown in Table 1. Among the 120 patients enrolled, the most common diagnosis was lumbar spinal stenosis (85 patients), followed by lumbar spondylolisthesis (21 patients), degenerative lumbar scoliosis (ten patients), and posterior revision surgery for failed lumbar degenerative disease (four patients). A total of 30 patients were included in each group. The mean age of patients in the ANH group, TXA group, ANH combined with TXA group, and control group were (58.43 ± 10.99) years, (59.77 ± 9.87) years, (59.04 ± 10.07) years, and (60.02 ± 9.01) years, respectively. The number of fused segments in the ANH group, TXA group, ANH combined with TXA group, and control group were 2.38 ± 0.91, 2.87 ± 1.01, 3.11 ± 0.99, and 2.99 ± 1.12, respectively. The number of interbody fusion in the ANH group, TXA group, ANH combined with TXA group, and control group were 1.28 ± 0.97, 1.34 ± 1.02, 1.61 ± 1.31, and 1.19 ± 1.13, respectively. No significant difference was found on the demographic parameters including patients’ age, gender, body weight, height, duration of surgery, body mass index, number of fused segments and interbody fusion in all the groups (*P* > 0.05).

**Table 1:** Demographic parameters and duration of surgery among different study groups

Parameters	ANH (*N* = 30)	TXA (*N* = 30)	ANH + TXA (*N* = 30)	Control (*N* = 30)	Overall *P* value
Age, years	58.43 ± 10.99	59.77 ± 9.87	59.04 ± 10.07	60.02 ± 9.01	0.679
Gender (female: male)	14:16	13:17	16:14	15:15	0.294
Body weight, kg	60.12 ± 4.65	59.88 ± 3.98	61.65 ± 5.07	61.96 ± 5.35	0.109
Height, cm	171.10 ± 6.03	170.67 ± 5.97	172.02 ± 6.01	171.69 ± 5.89	0.410
BMI	21.98 ± 2.27	21.69 ± 1.99	22.02 ± 2.02	21.42 ± 1.92	0.757
Duration of surgery, min	189.64 ± 35.89	195.64 ± 37.19	200.16 ± 36.97	185.85 ± 35.19	0.578
Number of fused segments	2.38 ± 0.91	2.87 ± 1.01	3.11 ± 0.99	2.99 ± 1.12	0.428
Number of interbody fusion	1.28 ± 0.97	1.34 ± 1.02	1.61 ± 1.31	1.19 ± 1.13	0.356

*BMI* body mass index, *ANH* Acute normovolemic hemodilution, *TXA* Tranexamic acid

Preoperative baseline hematological and biochemical parameters among the four study groups were compared. As shown in Table 2, no significant difference was found on hemoglobi, PCV, platelets, APTT, PT, and INR among groups (*P* > 0.05).

**Table 2:** Comparison of preoperative blood parameters among different study groups

Preoperative Variables	ANH	TXA	ANH + TXA	Control	Overall *P* value
Hemoglobin, g/dL	13.52 ± 1.53	12.99 ± 1.47	12.77 ± 1.59	13.02 ± 1.46	0.101
PCV	36.74 ± 3.97	37.93 ± 4.23	37.46 ± 4.35	38.27 ± 4.08	0.108
Platelets	2.98 ± 0.74	3.05 ± 0.81	3.01 ± 0.83	2.89 ± 0.77	0.701
APTT	27.45 ± 2.11	28.01 ± 3.07	27.07 ± 2.14	27.89 ± 2.65	0.379
PT	15.07 ± 2.05	14.98 ± 1.54	15.01 ± 1.61	15.19 ± 1.34	0.899
INR	1.01 ± 0.09	1.10 ± 0.19	1.08 ± 0.13	1.13 ± 0.17	0.501

*ANH* Acute normovolemic hemodilution, *TXA* Tranexamic acid, *APTT* activated partial thromboplastin time, *INR* international normalized ratio, *PCV* hematocrit or packed cell volume, *PT* prothrombin time

### Comparison of intraoperative blood loss, hemoglobin and PCV

Intraoperative blood loss and hemoglobin at the end of surgery among study groups were shown in Table 3. The mean intraoperative blood loss in the ANH group, TXA group, ANH combined with TXA group, and control group were (545.32 ± 189.29) mL, (378.72 ± 96.57) mL, (336.16 ± 88.464) mL, and (578.44 ± 201.81) mL, respectively. Intraoperative blood loss of the TXA group (*P* = 0.023) and the ANH combined with TXA group (*P* = 0.001) was statistically lower than those of the control group. Moreover, intraoperative blood loss in the ANH group was significantly higher than that in the TXA group (*P* = 0. 0029) and the ANH combined with TXA group (*P* = 0.009). No significant difference was found between the TXA group and the ANH combined with TXA group (*P* = 0.190). Meanwhile, no significant difference was detected between the ANH group and the control group (*P* = 0.082).

**Table 3:** Comparison of intraoperative blood loss and hematological indicators at the end of surgery among study groups

Intraoperative variables	ANH	TXA	ANH + TXA	Control	Overall *P* value
Blood loss, mL	545.32 ± 189.29	378.72 ± 96.57^ab^	336.16 ± 88.46^ab^	578.44 ± 201.81	< 0.001
Hemoglobin, g/dL (at the end of surgery)	12.07 ± 1.21	11.69 ± 1.37	11.94 ± 1.71	11.26 ± 1.26	0.195
PCV (at the end of surgery)	33.41 ± 4.12	32.67 ± 3.43	33.80 ± 4.89	31.98 ± 4.27	0.282
Hemoglobin, g/dL (at the postoperative first day)	10.13 ± 1.24^a^	9.98 ± 1.31^a^	11.07 ± 1.41^abc^	8.63 ± 1.63	0.154
PCV (at the postoperative first day)	30.98 ± 3.27^a^	31.89 ± 3.48^a^	33.68 ± 4.09^abc^	29.02 ± 3.06	0.161

Letter “a” indicated *P* < 0.05 compared with control group, letter “b” indicated *P* < 0.05 compared with ANH group, letter “c” indicated *P* < 0.05 compared with TXA group, respectively

*ANH* Acute normovolemic hemodilution, *TXA* Tranexamic acid, *PCV* packed cell volume

Hemoglobin and PCV at postoperative the first day in the ANH group (*P* = 0.019 and *P* = 0.032), TXA group (*P* = 0.025 and *P* = 0.032), and ANH combined with TXA group (*P* = 0.019 and *P* = 0.027) were significantly higher than those in the control group. Hemoglobin and PCV at postoperative the first day in the ANH combined with TXA group was statistically higher than those in the ANH group (*P* = 0.021 and *P* = 0.025) and TXA group (*P* = 0.043 and *P* = 0.041).

### Comparison of postoperative drain collection

Postoperative drain collection comparison among study groups was shown in Table 4. Mean postoperative drain collection in the ANH group, TXA group, ANH combined with TXA group, and control group were (360.00 ± 120.54) mL, (260.40 ± 109.58) mL, (240.00 ± 92.84) mL, and (410.00 ± 129.98) mL, respectively. Postoperative drain collection in the TXA group (*P* = 0.015) and the ANH combined with TXA group (*P* = 0.008) were significantly lower than those in the control group. Moreover, postoperative drain collection in the ANH group was significantly higher than that in the TXA group (*P* = 0.018) and the ANH combined with TXA group (*P* = 0.003). No significant difference was found between the TXA group and the ANH combined with TXA group (*P* = 1.003). Similarly, no significant difference was found between the ANH group and the control group (*P* = 0.778).

**Table 4:** Comparison of postoperative drain collection among study groups

	ANH	TXA	ANH + TXA	Control	Overall *P* value
Drain, mL	360.00 ± 120.54	260.40 ± 109.58^ab^	240.00 ± 92.84^ab^	410.00 ± 129.98	< 0.001

Letter “a” indicated *P* < 0.05 compared with control group, letter “b” indicated *P* < 0.05 compared with ANH group, respectively

*ANH* Acute normovolemic hemodilution, *TXA* Tranexamic acid

### Comparison of transfusion outcomes

Comparison of intraoperative and postoperative transfusion and rate of transfusion among study groups was shown in Table 5. Intraoperative transfusion amount and rate of intraoperative allogenic transfusion of the ANH group (*P* = 0.007 and *P* = 0.005), TXA group (*P* = 0.010 and *P* = 0.030), ANH combined with TXA group (*P* = 0.001 and *P* = 0.002) were statistically lower than those of the control group. Meanwhile, significant difference was found on intraoperative transfusion amount (*P* = 0.019, *P* = 0.012, and *P* = 0.024) and rate of intraoperative allogenic transfusion (*P* = 0.035, *P* = 0.027 and *P* = 0.014) between the ANH group and the TXA group, the ANH group and the ANH combined with TXA group, the TXA group and the ANH combined with TXA group.

**Table 5:** Comparison of intraoperative and postoperative transfusion amount and rate of transfusion among study groups

Transfusion	ANH	TXA	ANH + TXA	Control	Overall *P* value
Intraoperative allogenic transfusion amount, mL	182.21 ± 43.55^a^	251.21 ± 53.52^ab^	106.21 ± 32.55^abc^	447.21 ± 232.55	< 0.001
Intraoperative rate of allogenic transfusion, %	16.67^a^	33.33^ab^	6.67^abc^	63.33	< 0.001
Postoperative allogenic transfusion amount, mL	81.36 ± 13.11^a^	105.21 ± 14.69^ab^	43.12 ± 9.15^abc^	143.21 ± 24.57	< 0.001
Postoperative rate of allogenic transfusion, %	6.67^a^	13.33^ab^	3.33^abc^	20	< 0.001

Letter “a” indicated *P* < 0.05 compared with control group, letter “b” indicated *P* < 0.05 compared with ANH group, letter “c” indicated *P* < 0.05 compared with TXA group, respectively

*ANH* Acute normovolemic hemodilution, *TXA* Tranexamic acid

Similarly, postoperative transfusion amount and rate of postoperative allogenic transfusion of the ANH group (*P* = 0.037 and *P* = 0.01), TXA group (*P* = 0.039 and *P* = 0.013), ANH combined with TXA group (*P* = 0.006 and *P* = 0.002) was statistically lower than those in the control group. Meanwhile, significant difference was found on postoperative transfusion amount (*P* = 0.041, *P* = 0.022, and *P* = 0.013) and rate of postoperative allogenic transfusion (*P* = 0.03, *P* = 0.022 and *P* = 0.003) of the ANH group and TXA group, the ANH group and the ANH combined with TXA group, TXA group and the ANH combined with TXA group.

### Complications

No serious intraoperative or postoperative complication, such as dural tear, infection, epidural hematoma formation, deep-vein thrombosis, pulmonary embolism, allergic reaction, renal failure, or cardiopulmonary complications was found in all four groups. Furthermore, no minor side effects associated with the use of TXA such as nausea, vomiting, headache, or diarrhea occurred in all groups.

## Discussion

Lumbar spinal fusion surgery is one of the most commonly performed procedures of spine surgeries. There are various factors that either increase the risk of bleeding or require additional operative time in spine surgery, such as anatomical structure of spine, spongy vertebrae with its rich blood supply, and the fragile venous plexus wall that cannot self-contract after injury [17]. ANH and TXA were widely used in the spinal fusion surgery for blood management. However, the clinical outcomes remained controversial. Our data showed that intraoperative blood loss and postoperative drain collection of the TXA group and ANH combined with TXA group were significantly lower than those of the control group and ANH group. Intraoperative and postoperative transfusion amount and rate of intraoperative allogenic transfusion of the ANH group, TXA group, and ANH combined with TXA group were statistically lower than those of the control group. Hemoglobin and PCV at postoperative the first day in the ANH group, TXA group, and ANH combined with TXA group was significantly higher than those in the control group. The hemoglobin and PCV at postoperative first day in ANH combined with TXA group was statistically higher than the ANH group and TXA group. Thus, these data suggest implementation of ANH combined with TXA is an effective strategy for reducing the rate of transfusion in patients undergoing lumbar spinal fusion surgery.

Blood transfusion is crucial for radical surgery to avoid excessive bleeding. In past decades, ANH means blood withdrawal immediately before or after anesthetic induction, followed by dilution with colloids and/or crystalloids without reducing the circulating volume and then the circulating blood volume is supplemented with colloidal or crystal fluid and has been recommended to apply in surgeries with increased risk of bleeding [18]. ANH either achieves objective level of blood dilution, or avoids overloading circulation [10]. It could reduce the risk of hemolytic, allergic, pulmonary, immune-allergic reactions and acquisition of infectious-contagious diseases and can be obtained with a low operational cost [19]. In consistent with previous studies, we found no serious intraoperative or postoperative complications in all groups in this study.

TXA is a widely used synthetic fibrinolytic inhibitor. It can competitively inhibit the plasminogen activator and the adsorption of plasminogen to fibrin to prevent its activation and fibrin from being degraded by plasmin by binding lysine-binding sites of plasminogen. Moreover, it has the protective effect on platelets [20]. A dose of 15 mg/Kg TXA was intravenous injected at 15 min before skin incision was made followed by 1 mg/Kg/h infusion till skin closure in our study.

The intraoperative blood loss in the TXA group, and the ANH and TXA combination group were (378.72 ± 96.57) mL and (336.16 ± 88.464) mL, respectively, which was significantly lower than that in ANH group [(545.32 ± 189.29) mL] and control group [(578.44 ± 201.81) mL]. Though ANH could reduce the risk of complications, this method is associated with increased blood loss as a result of autologous blood being withdrawn. Therefore, the blood loss of ANH group was comparable with that of control group in this study. TXA can decrease bleeding by acting on the fibrinolytic system [21]. Elwatidy et al*.* [13] reported a significant reduction in blood loss in patients who were administered TXA during spine surgery. Yagi et al*.* [5] showed that the TXA group had significantly less intraoperative blood loss and postoperative blood loss during posterior spinal fusion for the treatment of idiopathic scoliosis in adolescents. Based on a randomized double-blind placebo controlled study, Shakeri et al. [22] showed that TXA could reduce both intraoperative and immediate postoperative blood loss, decrease the need for packed cell transfusion, and reduce the duration of hospitalization after complex spinal surgeries. Ni et al*.* demonstrated that intravenous TXA can effectively and safely reduce blood loss and bleeding-related complications after high tibial osteotomy and was beneficial for the blood management. Furthermore, data showed tendency toward less postoperative bleeding drain in TXA group [23].

The hemoglobin and PCV at the end of surgery were not significantly different among groups in this study. This result was partly in argument with previous study [19], which found reduced hemoglobin in ANH group as compared to control group. The hemoglobin and PCV at the postoperative first day was significantly higher in ANH group than that in control group. This can be attributed to the improved blood microcirculation of ANH [24, 25]. In addition, the autologous blood in ANH group preserves the platelet function and thus provides platelets and clotting factors after returning back to body [25]. We could also noticed that combined use of both ANH and TXA provided better improvement of hemoglobin and PCV at the post postoperative fist day.

The intraoperative allogenic transfusion amount and rate as well as the postoperative allogenic transfusion amount and rate were significantly decreased compared with those in control group. This might be associated with the less loss of red blood cell mass during surgery and the return of fresh platelets and clotting factors at the end of surgery. This result is consistent with most of the previous studies [25–28]. Moreover, intraoperative transfusion amount, allogenic transfusion, and postoperative transfusion amount of patients treated by TXA combined ANH were statistically lower than that in patients treated by ANH or TXA alone, respectively. Thus, it is reasonable to believe that the administration of TXA could improve the ability of ANH in reducing the blood loss, improving the recovery of hemoglobin and PCV and decreasing the need for allogenic blood transfusion.

However, there are some limitations in this study. First, the sample size of this study is relatively small. Only 30 patients were analyzed in each group. Second, the follow-up study was not conducted to compare the long-term outcomes of these four groups. Third, this study is a retrospective study, the information and data included are not sufficient and therefore subgroup analysis and multi-variate analysis were unable to perform.

## Conclusion

In conclusion, a combination of TXA and ANH might be a better choice for patients underwent lumbar spinal fusion surgery in reducing blood loss and the need for blood transfusion. However, further randomized double-blind study should be designed to verify the current conclusion.

## Data Availability

The data used to support the findings of this study are available from the corresponding author upon request.

## Abbreviations

ANH: Acute normovolemic hemodilution
TXA: Tranexamic acid
PCV: Packed cell volume
APTT: Platelets, activated partial thromboplastin time
PT: Prothrombin time
INR: International normalized ratio
ABL: Allowable blood loss

## References

[1] Elgafy H, Bransford RJ, McGuire RA, Dettori JR, Fischer D (2010). Blood loss in major spine surgery: Are there effective measures to decrease massive hemorrhage in major spine fusion surgery?. Spine (Phila Pa 1976).

[2] Winter SF, Santaguida C, Wong J, Fehlings MG (2016). Systemic and topical use of tranexamic acid in spinal surgery: a systematic review. Global Spine J.

[3] Sert GS, Cavus M, Kemerci P, Bektas S, Demir ZA, Ozgok A (2019). The results of cardiac surgery in terms of patient blood management in our hospital. Turk J Anaesthesiol Reanim.

[4] Visagie M, Qin CX, Cho BC, Merkel KR, Kajstura TJ, Amin RM (2019). The impact of patient blood management on blood utilization and clinical outcomes in complex spine surgery. Transfusion.

[5] Yagi M, Hasegawa J, Nagoshi N, Iizuka S, Kaneko S, Fukuda K (2012). Does the intraoperative tranexamic acid decrease operative blood loss during posterior spinal fusion for treatment of adolescent idiopathic scoliosis?. Spine (Phila Pa 1976).

[6] Jerico C, Osorio J, Garcia-Erce JA, Pera M (2019). Patient Blood Management strategies for iron deficiency anemia management in gastric cancer. Eur J Gastroenterol Hepatol.

[7] Spahn DR (2019). Patient blood management: What Else?. Ann Surg.

[8] Saricaoglu F, Akinci SB, Celiker V, Aypar U (2005). The effect of acute normovolemic hemodilution and acute hypervolemic hemodilution on coagulation and allogeneic transfusion. Saudi Med J.

[9] Carless PA, Henry DA, Moxey AJ, O'Connell D, Brown T, Fergusson DA (2010). Cell salvage for minimising perioperative allogeneic blood transfusion. Cochrane Database Syst Rev..

[10] Parasa SK, Bidkar PU, Parida S (2016). Acute normovolemic hemodilution to avoid blood transfusion during intracranial aneurysm surgery in a patient with atypical antibodies. Anesth Essays Res.

[11] Takekawa D, Saito J, Kinoshita H, Hashiba E, Hirai N, Yamazaki Y (2020). Acute normovolemic hemodilution reduced allogeneic blood transfusion without increasing perioperative complications in patients undergoing free-flap reconstruction of the head and neck. J Anesth.

[12] Yoo JS, Ahn J, Karmarkar SS, Lamoutte EH, Singh K (2019). The use of tranexamic acid in spine surgery. Ann Transl Med.

[13] Elwatidy S, Jamjoom Z, Elgamal E, Zakaria A, Turkistani A, El-Dawlatly A (2008). Efficacy and safety of prophylactic large dose of tranexamic acid in spine surgery: a prospective, randomized, double-blind, placebo-controlled study. Spine (Phila Pa 1976).

[14] Yang B, Li H, Wang D, He X, Zhang C, Yang P (2013). Systematic review and meta-analysis of perioperative intravenous tranexamic acid use in spinal surgery. PLoS ONE.

[15] Raksakietisak M, Sathitkarnmanee B, Srisaen P, Duangrat T, Chinachoti T, Rushatamukayanunt P (2015). Two doses of tranexamic acid reduce blood transfusion in complex spine surgery: a prospective randomized study. Spine (Phila Pa 1976).

[16] Soviero F, Geraci A, Termine S, Sanfilippo A, Maritano RM, D'Arienzo M (2010). Bleeding in orthopaedic surgery: the role of blood transfusion and erythropoietin alpha. Acta Biomed.

[17] Markowitz MA, Waters JH, Ness PM (2014). Patient blood management: a primary theme in transfusion medicine. Transfusion.

[18] Oppitz PP, Stefani MA (2013). Acute normovolemic hemodilution is safe in neurosurgery. World Neurosurg.

[19] Batista MFS, Costa CO, Vialle EN, Guasque J, Fiorentin JZ, Souza CS (2019). Acute normovolemic hemodilution in spinal deformity surgery. Rev Bras Ortop (Sao Paulo).

[20] Wong J, El Beheiry H, Rampersaud YR, Lewis S, Ahn H, De Silva Y (2008). Tranexamic acid reduces perioperative blood loss in adult patients having spinal fusion surgery. Anesth Analg.

[21] Benoni G, Fredin H (1996). Fibrinolytic inhibition with tranexamic acid reduces blood loss and blood transfusion after knee arthroplasty: a prospective, randomised, double-blind study of 86 patients. J Bone Joint Surg Br.

[22] Shakeri M, Salehpour F, Shokouhi G, Aeinfar K, Aghazadeh J, Mirzaei F (2018). Minimal dose of tranexamic acid is effective in reducing blood loss in complex spine surgeries: a randomized double-blind placebo controlled study. Asian Spine J.

[23] Peters A, Verma K, Slobodyanyuk K, Cheriyan T, Hoelscher C, Schwab F (2015). Antifibrinolytics reduce blood loss in adult spinal deformity surgery: a prospective, randomized controlled trial. Spine (Phila Pa 1976)..

[24] Milam JD, Austin SF, Nihill MR, Keats AS, Cooley DA (1985). Use of sufficient hemodilution to prevent coagulopathies following surgical correction of cyanotic heart disease. J Thorac Cardiovasc Surg.

[25] Bansal N, Kaur G, Garg S, Gombar S (2020). Acute normovolemic hemodilution in major orthopedic surgery. J Clin Orthop Trauma.

[26] Olsfanger D, Fredman B, Goldstein B, Shapiro A, Jedeikin R (1997). Acute normovolaemic haemodilution decreases postoperative allogeneic blood transfusion after total knee replacement. Br J Anaesth.

[27] Naqash IA, Draboo MA, Lone AQ, Nengroo SH, Kirmani A, Bhat AR (2011). Evaluation of acute normovolemic hemodilution and autotransfusion in neurosurgical patients undergoing excision of intracranial meningioma. J Anaesthesiol Clin Pharmacol.

[28] Goldberg J, Paugh TA, Dickinson TA, Fuller J, Paone G, Theurer PF (2015). Greater volume of acute normovolemic hemodilution may aid in reducing blood transfusions after cardiac surgery. Ann Thorac Surg..

